# Matrix-Assisted DOSY for Analysis of Indole Alkaloid Mixtures

**DOI:** 10.3390/molecules26061751

**Published:** 2021-03-20

**Authors:** Yu-Lin You, Fei-Fei Li, Ning Wang, Shu-Qi Wang

**Affiliations:** School of Pharmaceutical Sciences, Shandong University, 44# West Wenhua Road, Jinan 250012, China; youyulin@mail.sdu.edu.cn (Y.-L.Y.); lifeifei2013@126.com (F.-F.L.); wangningsdyxy@163.com (N.W.)

**Keywords:** NMR, DOSY, sodium dodecyl sulfate, indole alkaloid mixtures, diffusion resolution

## Abstract

Diffusion-ordered spectroscopy (DOSY) is a powerful tool for investigating mixtures and identifying peaks of chemical components. However, similar diffusion coefficients of the components, particularly for complex mixtures that contain crowded resonances, limit resolution and restrict application of the DOSY technique. In this paper, matrix-assisted DOSY were used to explore whether the diffusion resolution of a complex model involving indole alkaloid mixtures can be realized. Furthermore, we investigated the influence of different factors on the separation effect. The results showed that the changes in diffusion coefficient differences were achieved more obviously when using sodium dodecyl sulfate (SDS) micelles as the matrix. In addition, we also found that increasing the concentration of SDS can improve the resolution of the DOSY spectrum. Finally, after investigating the influence factors and NMR conditions, we demonstrated the applications of the SDS-assisted DOSY on analyzing the total alkaloid extract of *Alstonia Mairei*, and the virtual separation of mixtures was achieved.

## 1. Introduction

Alkaloids are an important class of nitrogen-containing organic compounds, widely existing in natural plants, and are pharmacologically active ingredients in many medicinal plants. There are various types of alkaloids, among which indole alkaloids are so far the most discovered, and are generally distributed in the *Apocynaceae*, *Loganiaceae*, and *Rubiaceae* families [1,2]. Indole alkaloids are complex in structure, and have significant biological activities [3,4,5], such as anticancer, anti-oxidation, and antibacterial. Clinically, they are generally used for anticancer [6], antidepressant [7], and neuronal protection [8]. *Alstonia mairei* is a plant of the genus *Alstonia* of *Apocynaceae*, which is rich in monoterpenoid indole alkaloids [9,10]. The species has been investigated with cleaning heat, relieving pain, hemostasis, and expelling pyogenic muscle [11]. In our previous studies, the ethanol extract from the branches and leaves of *Alstonia mairei* was found to have antitumor activity. However, we have not yet figured out which ingredient works, and this requires us to find a way to further study its chemical composition.

Many methods for the analysis of indole alkaloids have been developed and high-performance liquid chromatography (HPLC) is one of the most widely used methods [12,13]. However, due to the interaction between the basic nitrogen atom in the alkaloid structure and the un-bonded acidic silanol group of the stationary phase, this method has shortcomings, such as the broadening, tailing, and drifting of chromatographic peaks. In addition, the challenges to analyze the complex mixtures obtained from natural products are twofold: to achieve the effective separation of the components and to provide appropriate structural characteristics for each component [14]. NMR is a tool to precisely characterize structures of organic molecules. However, due to the structural similarity of the components in mixtures, the application of NMR spectroscopy in mixture research is limited and less common in this field [14]. Diffusion ordered spectroscopy (DOSY) is a special NMR method that can achieve the purposes of qualitative analysis of the mixtures. DOSY is a pseudo two-dimensional spectrum, one dimension being a chemical shift, and the other dimension a diffusion coefficient. The line width of the spectral peak in the diffusion coefficient dimension is determined by the standard deviation of the fit [15]. Different diffusion behaviors are utilized to distinguish NMR signals of different species in DOSY experiments. Its basic form relies on differences in the hydrodynamic radius to distinguish mixtures of different diffusion coefficients [16]. Compared with traditional separation technology, DOSY can analyze the components in the mixture without physical separation. Therefore, this technology has unique advantages in the separation and identification of natural product mixtures [17]. At present, the DOSY experiment can be applied to the analysis of mixtures according to the diffusion speed of the corresponding molecules in the peaks of the one-dimensional spectrum along the diffusion coefficient axis [18,19]. In addition, by measuring the diffusion coefficients of molecules in a solution, the interaction between molecules can be characterized according to changes in the diffusion coefficients, including the characterization of reaction intermediates [20], revealing different submicellar solubilization mechanisms [21], enantiodiscrimination [22], and measuring protein size [23] and carbohydrate molecular weight [24]. However, it is difficult to analyze a mixture of similar species by conventional DOSY technology.

In liquid chromatography, the translation speed of each component of mixtures can be selectively changed by interacting with the stationary phase [25,26]. Research has found that adding a conventional stationary phase used in HPLC as a matrix to the NMR tube, such as silica gel, can enhance the separation performance of the NMR method and achieve the purpose of differentiation [27,28,29,30,31,32,33]. The reason is that the interaction between the matrix and analytes can be used to enlarge the apparent diffusion coefficient differences between similar analyte molecules.

From the reported literature [34], DOSY currently shows good separation performance for simple mixture systems, but for alkaloid mixtures, which are complex in structure, there are relatively few studies in this area. Therefore, in this experiment, we designed a simulated natural alkaloid extract model system consisting of four indole alkaloids to prove the application of DOSY in the separation of indole alkaloid mixtures.

## 2. Results and Discussion

### 2.1. Evaluation of Different Matrices to Separate Indole Alkaloid Mixtures

**Figure 1:**
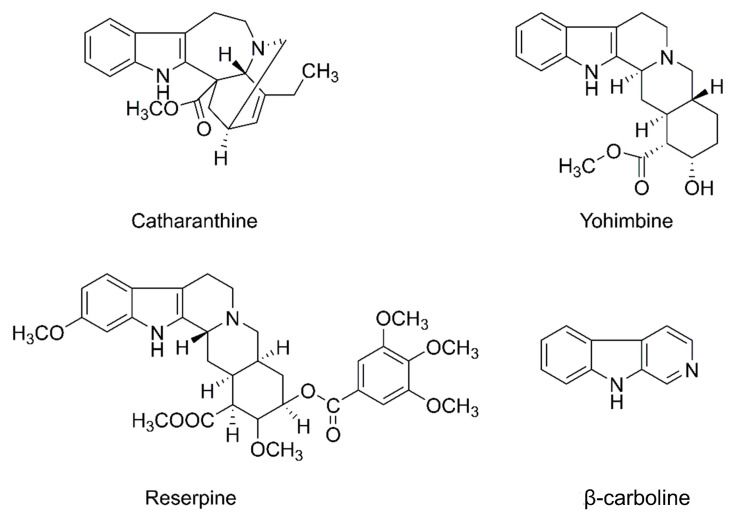
Structures of indole alkaloids used in this study.

In the DOSY experiments, the structures of additional substances adding to the system are supposed to be as simple as possible to avoid their overlapping with the analyte signals on the spectrum [32]. A series of matrices, including polydimethylsiloxane (PDMS), sodium dodecyl sulfate (SDS), polyvinylpyrrolidone (PVP), and polyethyleneglycol (PEG), were selected, and the mixtures of reserpine, catharanthine, β-carboline, and yohimbine were then selected as analytes. The structural formulas of the four indole alkaloids are shown in Figure 1. DMSO-*d*_6_ was used as the NMR solvent.

When conducting the DOSY experiments, the crucial condition is that the diffusion coefficient (D) of each analyte is different. It can be seen from the published application research literature that most of the relative change values of the diffusion coefficients are in the tens of percentage points, and a few are only a few percent [35,36,37]. Furthermore, studies have shown that the DOSY experiment can resolve changes in the diffusion coefficient less than 2% [36]. In order to better present the experimental results, we used the following formula [38] to evaluate its separation effect:

(1) $$\Delta D=D_{A1}-D_{A2}$$

where ΔD is the degree of separation between components, D_A1_ is the diffusion coefficient of analyte 1 (A1), and D_A2_ is the diffusion coefficient of analyte 2 (A2).

The results and the data related to the model were plotted as a graph in order to analyze the results intuitively.

**Figure 2:**
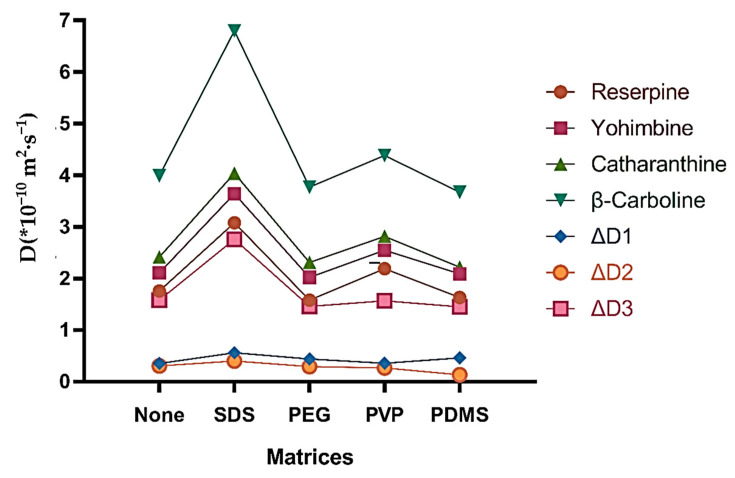
The influence of different matrices on the diffusion coefficient and resolution. Matrices and mixtures (each component was 2 mg) were dissolved in 600 μL of DMSO-*d*_6_ when T was set at 298 K.

Figure 2 shows the influence of different matrices on D and ΔD values. As we can see from Figure 2, the D values of the four analytes changed with the addition of matrices. ΔD_1_ (D_yohimbine_ − D_reserpine_), ΔD_2_ (D_catharanthine_ − D_yohimbine_), and ΔD_3_ (D_β-carboline_ − D_catharanthine_) changed the most with SDS, which revealed that the components were better resolved with SDS. The ^1^H DOSY spectra (Figure S1) and the specific value of D and ΔD (Table S1) are reported in the Supplementary Materials section.

As reported, it has been proved that surfactants can be used as co-solvents to systematically control the diffusion resolution and change the diffusion behavior of substances in DOSY experiments [34]. Based on this consideration and the experiments we have done, SDS was selected as the most suitable matrix in this work.

**Figure 3:**
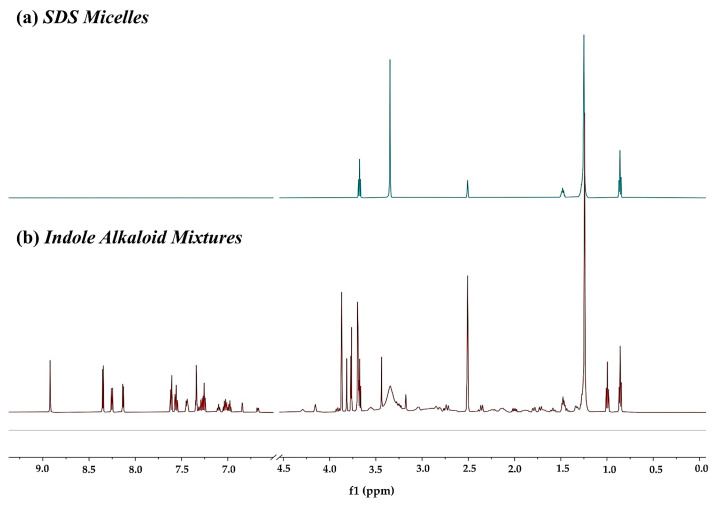
^1^H-NMR spectra at 600 MHz of (**a**) sodium dodecyl sulfate (SDS) and (**b**) indole alkaloid mixtures with SDS.

**Figure 4:**
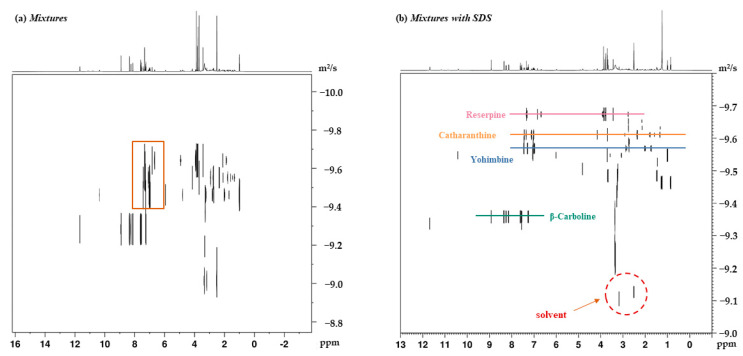
(**a**) DOSY spectrum of mixtures (each component was 2 mg) dissolved in 600 μL of DMSO-*d*_6_. (**b**) DOSY spectrum of the same mixtures as in (**a**), but in the presence of 2 mg of SDS.

Figure 3 shows the ^1^H NMR spectrum of mixtures mixed with SDS. In a mixture consisting of the aforementioned indole alkaloids and SDS micelles, the peaks of an addictive structure overlap with those of the analytes around 0.86, 1.47, and 3.67 ppm, which avoids affecting the aromatic region that contains the signals of interest. Figure 4 displays the ^1^H DOSY spectra of indole alkaloid mixtures and demonstrates the effects on the diffusion coefficient by introducing a surfactant. As expected, the signals of the four analytes were not completely separated without a matrix, since the diffusion coefficients were too close to achieve sufficient separation on the diffusion dimension. By contrast, the four component signals were fully separated in DOSY experiments after the addition of SDS. At the same time, the diffusion coefficients of the four analytes increased to varying degrees. Among the four analytes, reserpine had the largest molecular weight, followed by yohimbine, catharanthine, and, finally, β-carboline. From the Stokes–Einstein equation, the molecular self-diffusion coefficient is proportional to the molecular hydrodynamic radius inversely. The results also confirmed that, among the four analytes, reserpine had the smallest self-diffusion coefficient, catharanthine and yohimbine had the middle self-diffusion coefficient, and β-carboline had the largest self-diffusion coefficient.

### 2.2. Optimization of DOSY Experimental Conditions

**Figure 5:**
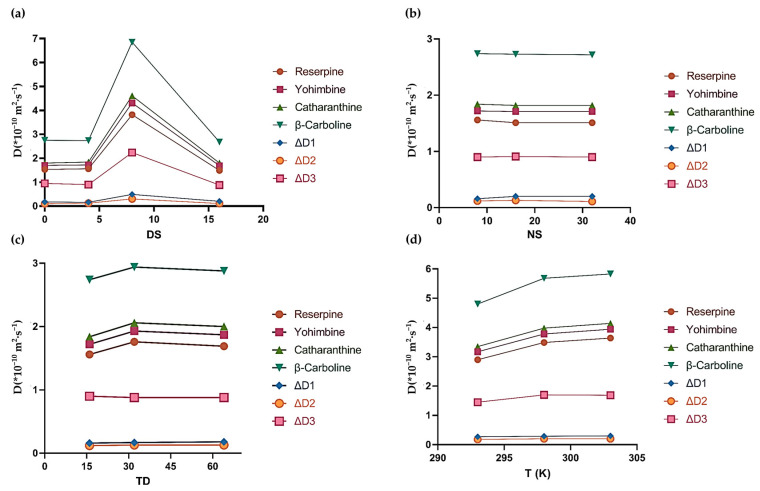
(**a**) The influence of a DS on the diffusion coefficient and resolution. The DS was set as 0, 4, 8, and 16. (**b**) The influence of NS on the diffusion coefficient and resolution. NS was set as 8, 16, and 32. (**c**) The influence of TD on the diffusion coefficient and resolution. TD was set as 16, 32, and 64. (**d**) The influence of experimental temperature (T) on the diffusion coefficient and resolution. T was set as 293 K, 298 K, and 303 K. Each component was 2 mg. DMSO-*d*_6_ was chosen as the solvent.

The number of dummy scans (DS) is an important parameter in DOSY experiments. The influence of the DS lies in whether a balanced and stable condition can be reached before the experiment sampling, and whether it will affect the results of the experiments when the time is insufficient. We set the DS as 0, 4, 8, and 16. As we can see from Figure 5a, the D values of the four analytes changed. Regarding the variation of ΔD values we are concerned about, the results showed that ΔD significantly reached the maximum when the DS was set as 8 (Table S2). This phenomenon demonstrates that using a matrix to separate components in the mixtures was inseparable from the existence of a DS, but higher DS values may not achieve a better result.

The number of scans (NS) is one of the main parameters that has an impact on the signal-to-noise ratio (S/N) of the spectrum. The S/N of the spectrum directly affects the experimental data obtained by subsequent processing. In the present study, the DOSY experiments were conducted using different numbers of scans (NS = 8, 16, and 32). From the results shown in Figure 5b, the NS has a little impact on the experimental results (Table S3). Furthermore, the NS is also related to the sample concentration of the experiment itself. In the case of high concentration, a smaller NS can be used for sampling. When we carried out an experiment, a value of 16 was selected according to actual needs.

In addition, the values of the FID data points (TD) are the data points used in the final data processing. Thus, we used 16, 32, and 64 for experiments. In the data we obtained in Figure 5c, increasing TD had no obvious optimization effect on the spectrum, and what followed was increased experimental time (Table S4). In existing DOSY experiments, the default of TD was 16. Considering this, the TD of the indirect dimension was set as 16 in the subsequent experiments.

According to the Stokes–Einstein equation, temperature can also affect the diffusion coefficient of the component. However, the solvent DMSO-*d*_6_ will freeze at a temperature of 288 K, which precludes the test from being completed. Thus, we set the temperature at 293 K, 298 K, and 303 K to investigate the effect of temperature on the separation effect of SDS-assisted DOSY. It can be seen in Figure 5d that, as the temperature increased, the diffusion coefficients of the four components in the mixture gradually increased between 293 and 303 K, but were almost unchanged when the temperature was between 298 and 303 K (Table S5). Moreover, convection occurs when a temperature gradient occurs, even if the temperature gradient is 0.2 K. The introduction of convection greatly affects the repeatability of the experiment [39], so we used 298 K in the following experiments.

Table 1 summarizes all examined parameters and their variations, and the best values were presented in bold in the table.

### 2.3. Investigation of the Influence Factors on the Separation Performance

#### 2.3.1. Influence of SDS Concentration

**Figure 6:**
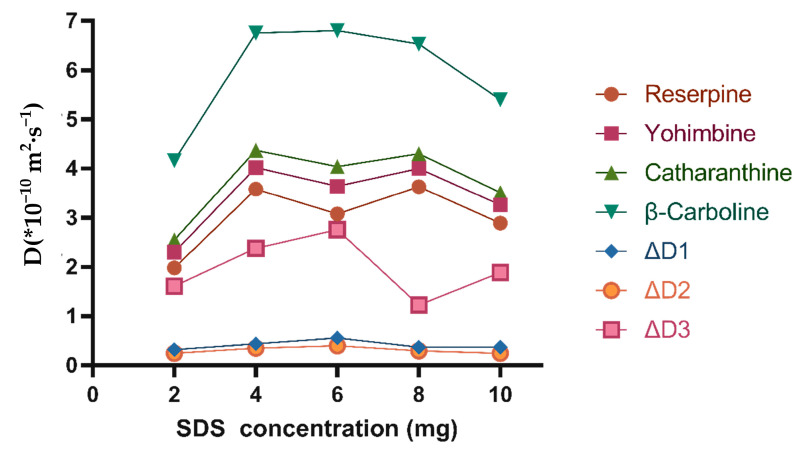
The influence of SDS concentration on the diffusion coefficient and resolution. Mixtures (each component was 2 mg) and SDS (2 mg, 4 mg, 6 mg, 8 mg, and 10 mg) were dissolved in 600 μL of DMSO-*d*_6_ when T was set at 298 K.

Figure 6 shows the influence of the SDS concentration on D and ΔD values. As can be seen in Figure 6, the diffusion coefficients of the analytes exhibited a dramatic change with the increase in the SDS concentration, but when the quantity of SDS in the solution reached 6 mg, the ΔD began to decrease. The presence of SDS made the diffusion coefficients of the four analytes increase. Among them, the diffusion coefficients of catharanthine and yohimbine with similar molecular weight were nearly 20% different (Table S6). In addition, as can be seen from Figure S2 reported in the Supplementary Materials section, we can conclude that there is no interaction between the analytes and SDS, so it can be inferred that the resolution of the analytes in the mixture only slightly increases with the increase in viscosity. However, too much SDS added in the experiment will bring out a higher viscosity of the solvent, and excessive viscosity may make it difficult for the component molecules in the solution to diffuse, thus affecting the diffusion coefficient of each component in the mixture. Therefore, the ΔD value shows a downward trend.

#### 2.3.2. Influence of Solvent Polarity

The solvent is an important factor affecting chromatographic behavior. The polarity of the solvent affects the presence of sample molecules and further affects its resolution. We selected the deuterated chloroform (CDCl_3_), which is quite different in polarity from DMSO-*d*_6_, to investigate the effect of solvent polarity on the DOSY separation performance.

**Figure 7:**
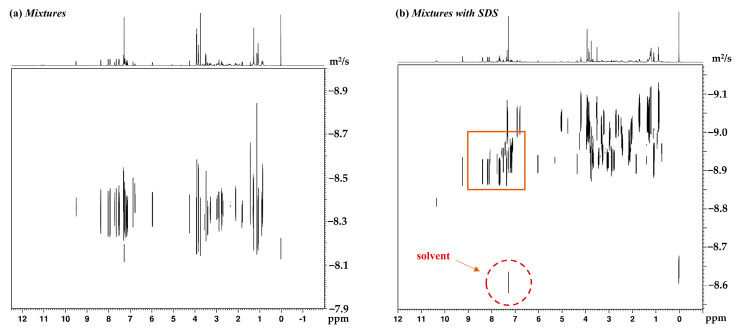
(**a**) DOSY spectrum of mixtures (each component was 2 mg) dissolved in 600 μL of CDCl_3_. (**b**) DOSY spectrum of the same mixtures as in (**a**), but in the presence of SDS.

The influence of the polarity of the solvent on ΔD values is shown in Figure 7. We found that the phenomena observed in DMSO-*d*_6_ and CDCl_3_ were quite different, and the separation effect of the four components in DMSO-*d*_6_ was stronger than that of CDCl_3_. For DMSO-*d*_6_, the change in the diffusion coefficient of the analyte molecule is mainly caused by the viscosity effect and the hydrogen bonding between the analyte and the solvent. When the test temperature was 298 K, the viscosity coefficients of CDCl_3_ and DMSO-*d*_6_ were 0.54 mPa·s and 1.987 mPa·s, respectively. When DMSO-*d*_6_ was used as the solvent, the corresponding solution viscosity increased slightly, and the resolution increased with higher viscosity. Furthermore, as a dipolar protic solvent, DMSO-*d*_6_ has a weak tendency to accept protons, and the ability to form hydrogen bonds, while CDCl_3_ as an inert solvent has no such ability. The presence of the hydrogen bonding force between the analyte and DMSO-*d*_6_, makes the change in the ΔD value more obvious, and well-resolved (in diffusion dimensions) DOSY spectra was seen. Finally, the low solubility of analytes in CDCl_3_ also limits the signal-to-noise ratio of ^1^H spectra and leads to a poorer resolution in the diffusion domain of the DOSY spectra. Therefore, the polarity of the solvent not only affects the separation results of the DOSY experiment, but also changes the separation performance of DOSY. 

### 2.4. The Application of SDS-Assisted DOSY in Separation of Total Alkaloid Extract

Through the experimental study of the above model compounds, it can be known that DOSY technology has good separation ability for alkaloids, and can realize virtual separation of components in the mixtures. On the basis of applying the DOSY technology to the theoretical model mixture, we introduced the technology into the actual mixture system, using the total alkaloid extract of *Alstonia Mairei* as the analyte to explore the value of SDS-assisted DOSY in practical application.

**Figure 8:**
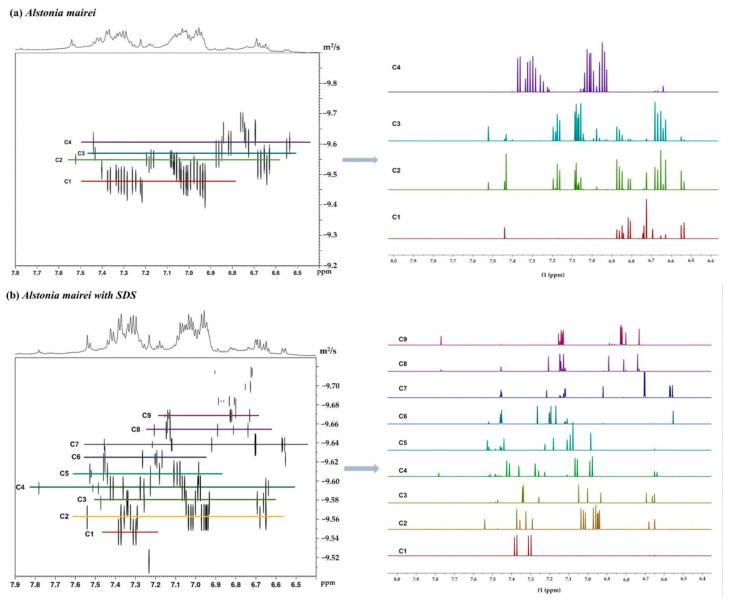
DOSY spectra of a mixture of the real total alkaloid extract (20 mg) before (**a**, upper) and after (**b**, lower) the addition of SDS (10 mg) in DMSO-*d_6_* (600 μL). Experimental temperature was 298 K.

The DOSY experiment results of the total alkaloid extract sample of *Alstonia Mairei* are shown in Figure 8. It was obvious that the diffusion resolution in the total alkaloid extract without SDS was limited in the diffusion dimension. Based on the DOSY spectrum alone, it was impossible to accurately assign the proton signal peaks for each compound. Furthermore, since the chemical shifts of different protons in the components were similar, it may cause the overlap of the proton signal peaks. It was apparent that, after the addition of SDS, the diffusion resolution of the mixture was greatly improved, and the diffusion coefficients between the components changed greatly in the DOSY spectrum of the mixture. From the extracted hydrogen spectrum, it also can be seen that the overlap in the aromatic region was small, which was more conducive to the next analysis. The experimental results show that it is feasible to apply DOSY technology to the total alkaloid extract mixture system, and provide a theoretical guidance basis for further qualitative and quantitative analysis of the components in the mixture.

In the previous work of this research group, the ^1^H chemical shift data of natural products was collected from the literature, and has been used to establish a large experimental database. More than 200 indole alkaloids representing *Apocynaceae* were included in this experimental database. These alkaloids were selected because of the diversity of their structures. For some compounds, the ^1^H spectra of which were not recorded in the literature, we used the NMRP module in the MestReNova software to make predictions in order to enrich the database. As a routine verification test, the MestReNova software was used to search ^1^H spectra extracted from the DOSY spectrum of the *Alstonia mairei* mixtures, and then the results were ranked by accuracy. In order to make the results more reliable, we used the search results of the ^1^H spectra extracted from the model mixture with SDS (Table S8) as a control. The option with the highest score was considered to be the compound we separated. Moreover, compared with the score of the experiment without SDS, it was obvious that the addition of SDS matrix improved the resolution of DOSY spectra. In the DOSY spectrum, the analyte signals overlapped due to the similar structures of analytes. In a practical sample without SDS, the signals of the alkaloid extract overlapped closely, and only four ^1^H-NMR spectra could be extracted from the DOSY spectrum. After adding the SDS matrix, the strong overlapping areas were separated. Nine ^1^H-NMR spectra were extracted from the DOSY spectrum of the alkaloid-SDS mixture. The results were recorded in the Supplementary Materials section (Table S9). Therefore, the SDS matrix could be used to improve the resolution of the DOSY spectrum.

## 3. Materials and Methods

### 3.1. Ragents and Materials

The following reagents and materials were obtained: dimethyl sulfoxide-*d*_6_ (DMSO-*d*_6_, 99.9%, Cambridge Isotope Laboratories, Inc., Andover, Massachusetts, United States), reserpine (≥98%, HPLC grade, Shanghai Macklin Biochemical Co., Ltd., Shanghai, China), catharanthine (≥95%, Energy Chemical, Shanghai, China), β-carboline (≥98%, Meryer Shanghai Chemical Technology Co., Ltd., Shanghai, China), yohimbine (≥99%, Shanghai Aladdin Bio-Chem Technology Co., Ltd., Shanghai, China), polydimethylsiloxane (PDMS, FEIYUBIO, Nantong, China), sodium dodecyl sulfate (SDS, Solarbio), polyvinyl pyrrolidone (MW 10,000, Shanghai Macklin Biochemical Co., Ltd., Shanghai, China), and polyethylene glycol (MW 4000, Shanghai Macklin Biochemical Co., Ltd., Shanghai, China).

### 3.2. Instrumentation

The following instrumentation was utilized: a BRUKER AVANCE 600 MHz NMR spectrometer consisting of an ultra-low temperature probe (BRUKER, Fällanden, Switzerland), a BSA 124S electronic analytical balance (Satorius, AG, Goettingen, Germany), and an SB-4200 DTD ultrasonic cleaning machine (Ningbo Xinzhi Biotechnology Co., Ltd., Ningbo, China).

### 3.3. NMR Experiments

Routine DOSY NMR experiments were carried out using the BRUKER-AV600 NMR instrument equipped with a 5 mm ^1^H/^13^C BBO probe. The diffusion measurements were recorded using an LEDBPG2S pulse sequence, ramping the strongest gradient from 2% to 95% of maximum strength in 30 steps. All ^1^H-NMR spectra were obtained at a spectral width of 12,019.230 Hz, and the number of acquired complex data points was 4096. Diffusion experiments were advanced using a 0.6 ms spoil gradient duration, a 1 ms relaxation delay, and a 5 ms eddy current recovery delay. The experimental temperature was set at 298 K, except for specific testing requirements. The diffusion times and gradient pulse duration were optimized in subsequent experiments according to practical needs.

The data of the mixtures were apodized by a sine function and zero-filled up to 65,536 complex data points [29]. With regard to the Fourier transformation, phase adjustment, and baseline correction, the diffusion dimension was processed by Bruker Topspin 3.6.2 spectrometer operating software. Finally, two-dimensional DOSY spectra were further analyzed by BRUKER Dynamics Center 2.6.2 and MestReNova 14.2.0 software.

## 4. Conclusions

In summary, the DOSY NMR method for analysis of an indole alkaloid mixture was developed in this study, and we carried out a more in-depth study in conjunction with the SDS micelles reported in the literature. The results showed that the components in the model mixture can be virtually separated with the addition of a matrix. Subsequently, we optimized the NMR conditions and determined the best experimental conditions with the DS as 8, NS as 16, and TD as 16. In addition, several influencing factors were investigated. Among them, increasing the concentration of SDS and increasing the detection temperature will increase the resolution, and the polarity of the solvent can change the separation effect of SDS-assisted DOSY. Finally, the suitability of the NMR method was eventually evaluated on a real sample. We used the total alkaloid extract of *Alstonia mairei* as the actual sample, and used the SDS-assisted DOSY technology to analyze the components in the mixture. The experimental results show that it is feasible to apply this technology to the indole alkaloid mixture system.

The SDS-assisted DOSY NMR method described in this study offers a new approach for practical use in the analysis of complex mixtures of natural products. Without the need for physical separation, mixtures can be analyzed using peak separation, which ensures that the original chemical environment in the mixture system is not destroyed, thereby obtaining more effective information. To summarize, this lays a foundation for further qualitative and quantitative analyses of the components.

## Figures and Tables

**Table 1:** Summary of all of the examined parameters and their variation ranges.

No.	Parameters	Values
1	Number of dummy scans	0, 4, 8, **16** ^a^
2	Number of scans	**8**, 16, 32
3	The FID data points	**16**, 32, 64
4	Experimental temperature (K)	293, **298**, 303

^a^ Bold values represents the best parameter values of the experimental.

## Data Availability

The data presented in this study are available on request from the corresponding author.

## Supplementary Materials

The supplementary materials are available online.

## Sample Availability

Samples of the compounds are available from the authors.
