# Supplementary material for: Matrix-Assisted DOSY for Analysis of Indole Alkaloid Mixtures
Source: Molecules. 2021 Mar 20;26(6):1751. doi: 10.3390/molecules26061751 (PMC8003906; doi:10.3390/molecules26061751)
Supplement: Supplementary file 1 [file molecules-26-01751-s001.pdf]

Supplementary Materials

Matrix-Assisted DOSY for Analysis of Indole Alkaloid Mixtures

Yu-Lin You, Fei-Fei Li, Ning Wang and Shu-Qi Wang *

School of Pharmaceutical Sciences, Shandong University, 44# West Wenhua Road, Jinan 250012, China

youyulin@mail.sdu.edu.cn (Y.-L.Y.); lifeifei2013@126.com (F.-F.L.); wangningsdyxy@163.com (N.W.)

* Correspondence: wangsq@sdu.edu.cn; Tel./Fax: +86-0531-88382014

Table of contents

**Figure S1**.	Experimental results for an indole alkaloid mixture of reserpine, catharanthine, β-carboline and yohimbine in DMSO-d_6.	3
**Figure S2.**	^{1}H NMR spectra at 600 MHz of (a) indole alkaloid mixtures (each component was 2 mg) and (b) the same mixtures as in (a), but in the presence of SDS. Signals from SDS micelles were marked by red dot.	4
**Table S1.**	Influence of different matrices on the diffusion coefficient and resolution.	**5**
**Table S2.**	Influence of the number of dummy scans (DS) on diffusion coefficient and resolution.	**6**
**Table S3.**	Influence of the number of scans (NS) on diffusion coefficient and resolution.	**7**
**Table S4.**	Influence of the values of the FID data points (TD) on diffusion coefficient and resolution.	**8**
**Table S5.**	Influence of experimental temperature (T) on diffusion coefficient and resolution.	**9**
**Table S6.**	Influence of SDS concentration (C) on diffusion coefficient and resolution.	**10**
**Table S7.**	Influence of the sample stability on diffusion coefficient and resolution.	**11**
**Figure S3.**	DOSY spectrum of model mixtures (each component was 2 mg) in the presence of 2 mg SDS dissolved in 600 μL of DMSO-d_6.	**13**
**Table S8.**	Searching ^{1}H spectrum extracted from the DOSY spectrum of the model mixture in the database, and the following are the options with the highest scores and their corresponding compounds.	**13**
**Figure S4.**	DOSY spectra of a mixture of the real total alkaloid extract (20 mg) before (a, upper) and after (b, lower) the addition of SDS (10 mg) in DMSO-d6 (600 μL). Experimental temperature was 298 K.	**14**
**Table S9.**	Searching ^{1}H spectrum extracted from the DOSY spectrum of the actual sample mixture in the database, and the following are the options with the highest scores and their corresponding compounds.	**14**

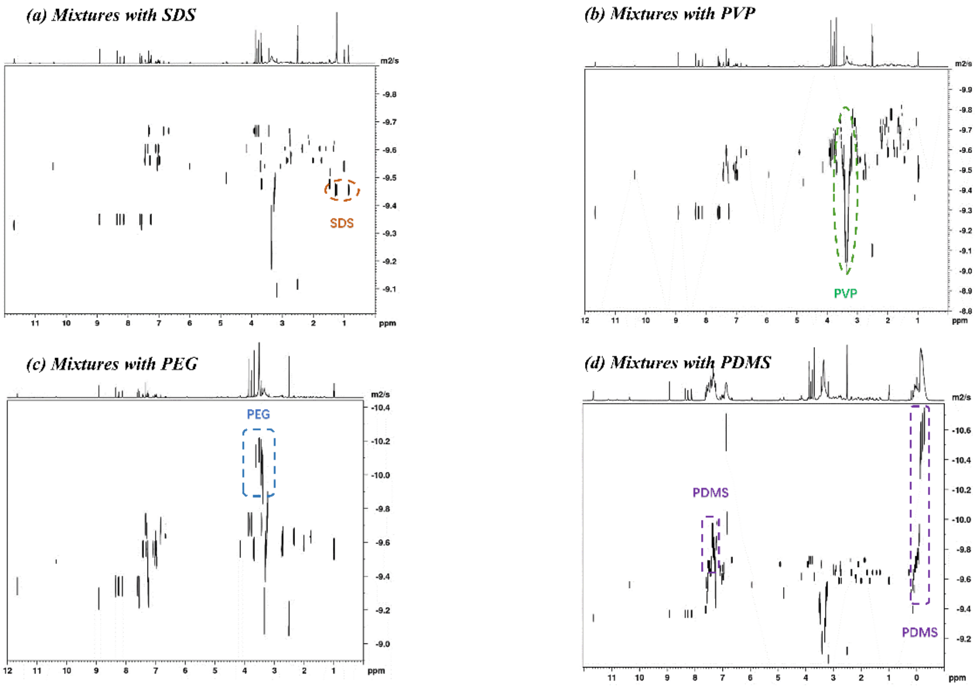

Fig. S1. Experimental results for an indole alkaloid mixture of reserpine, catharanthine, *β*-carboline and yohimbine in DMSO-d_6. 2D DOSY spectral of (a)mixtures with SDS, (b) mixtures with PVP, (c) mixtures with PEG, (d) mixtures with PDMS were obtained. Signals from different matrices were marked by circles of different colors. Diffusion coefficient (D) was reported in $m^2s^{-1}\times10^{-10}$.

Figure S2. ^{1}H NMR spectra at 600 MHz of (***a***) indole alkaloid mixtures (each component was 2 mg) and (***b***) the same mixtures as in (***a***), but in the presence of SDS. Signals from SDS micelles were marked by red dot.

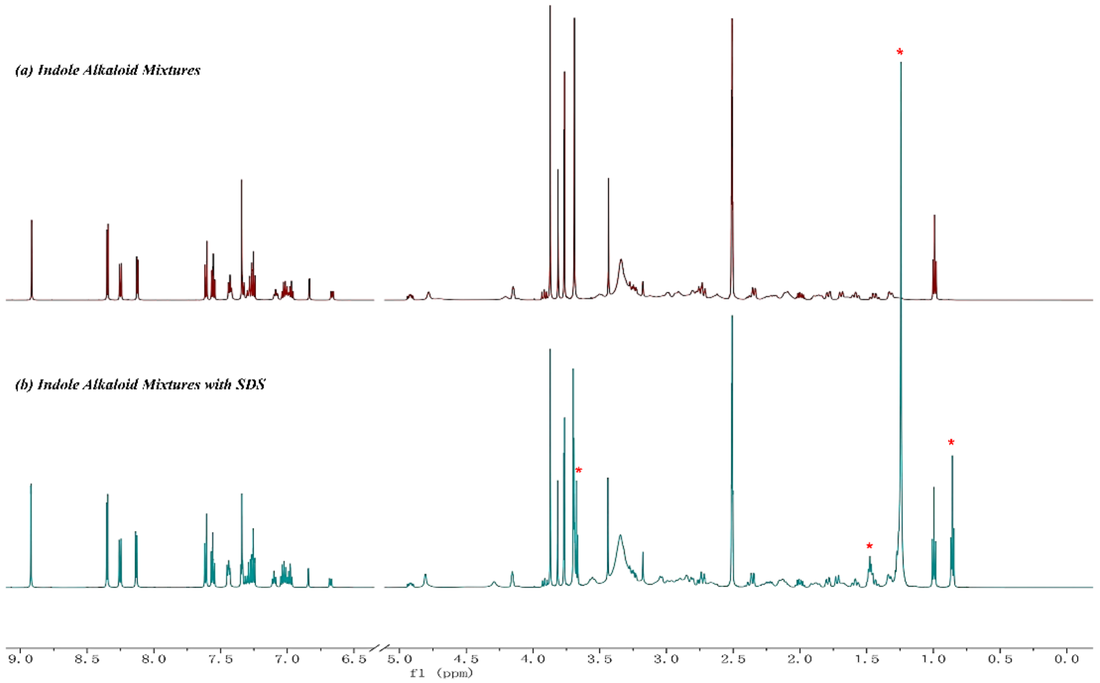

Table S1. Influence of different matrices on the diffusion coefficient and resolution.

	reserpine	yohimbine	catharanthine	β-carboline	ΔD1	ΔD2	ΔD3
**None**	1.76	2.11	2.41	3.99	0.35	0.30	1.58
**SDS**	3.08	3.64	4.04	6.80	0.56	0.40	2.76
**PEG**	1.58	2.02	2.31	3.77	0.44	0.29	1.46
**PVP**	2.19	2.55	2.81	4.38	0.36	0.27	1.57
**PDMS**	1.63	2.09	2.22	3.67	0.46	0.13	1.45

$\Delta D_1 = D_{yohimbine} - D_{reserpine}$, $\Delta D_2 = D_{catharanthine} - D_{yohimbine}$, $\Delta D_3 = D_{\beta\text{-carboline}} - D_{catharanthine}$

Diffusion coefficient (D) was reported in $m^2s^{-1}\times10^{-10}$.

Table S2. Influence of the number of dummy scans (DS) on diffusion coefficient and resolution.

DS	reserpine	yohimbine	catharanthine	β-carboline	ΔD1	ΔD2	ΔD3
**0**	1.53	1.70	1.80	2.75	0.17	0.10	0.95
**4**	1.56	1.72	1.84	2.74	0.16	0.12	0.90
**8**	3.82	4.31	4.61	6.85	0.49	0.30	2.24
**16**	1.49	1.69	1.80	2.68	0.20	0.11	0.88

$\Delta D_1 = D_{yohimbine} - D_{reserpine}$, $\Delta D_2 = D_{catharanthine} - D_{yohimbine}$, $\Delta D_3 = D_{\beta\text{-carboline}} - D_{catharanthine}$

Diffusion coefficient (D) was reported in $m^2s^{-1}\times10^{-10}$.

TableS3. Influence of the number of scans (NS) on diffusion coefficient and resolution.

NS	reserpine	yohimbine	catharanthine	β-carboline	ΔD1	ΔD2	ΔD3
**8**	1.56	1.72	1.84	2.74	0.16	0.12	0.90
**16**	1.51	1.71	1.82	2.73	0.20	0.13	0.91
**32**	1.51	1.71	1.82	2.72	0.20	0.11	0.90

$\Delta D_1 = D_{yohimbine} - D_{reserpine}$, $\Delta D_2 = D_{catharanthine} - D_{yohimbine}$, $\Delta D_3 = D_{\beta\text{-carboline}} - D_{catharanthine}$

Diffusion coefficient (D) was reported in $m^2s^{-1}\times10^{-10}$.

TableS4. Influence of the values of the FID data points (TD) on diffusion coefficient and resolution.

TD	reserpine	yohimbine	catharanthine	β-carboline	ΔD1	ΔD2	ΔD3
**16**	1.56	1.72	1.84	2.74	0.16	0.12	0.90
**32**	1.76	1.93	2.06	2.94	0.17	0.13	0.88
**64**	1.69	1.87	2.00	2.88	0.18	0.13	0.88

$\Delta D_1 = D_{yohimbine} - D_{reserpine}$, $\Delta D_2 = D_{catharanthine} - D_{yohimbine}$, $\Delta D_3 = D_{\beta\text{-carboline}} - D_{catharanthine}$

Diffusion coefficient (D) was reported in $m^2s^{-1}\times10^{-10}$.

Table S5. Influence of experimental temperature (T) on diffusion coefficient and resolution.

T (K)	reserpine	yohimbine	catharanthine	β-carboline	ΔD1	ΔD2	ΔD3
**293 K**	2.90	3.17	3.35	4.80	0.27	0.18	1.45
**298 K**	3.49	3.78	3.98	5.68	0.29	0.20	1.70
**303 K**	3.64	3.94	4.14	5.83	0.30	0.20	1.69

$\Delta D_1 = D_{yohimbine} - D_{reserpine}$, $\Delta D_2 = D_{catharanthine} - D_{yohimbine}$, $\Delta D_3 = D_{\beta\text{-carboline}} - D_{catharanthine}$

Diffusion coefficient (D) was reported in $m^2s^{-1}\times10^{-10}$.

Table S6. Influence of SDS concentration (C) on diffusion coefficient and resolution.

C	reserpine	yohimbine	catharanthine	β-carboline	ΔD1	ΔD2	ΔD3
**2mg**	1.98	2.30	2.55	4.16	0.32	0.25	1.61
**4mg**	3.58	4.02	4.37	6.75	0.44	0.35	2.38
**6mg**	3.08	3.64	4.04	6.80	0.56	0.40	2.76
**8mg**	3.63	4.00	4.30	6.53	0.37	0.30	1.23
**10mg**	2.89	3.26	3.51	5.40	0.37	0.25	1.89

$\Delta D_1 = D_{yohimbine} - D_{reserpine}$, $\Delta D_2 = D_{catharanthine} - D_{yohimbine}$, $\Delta D_3 = D_{\beta\text{-carboline}} - D_{catharanthine}$

Diffusion coefficient (D) was reported in $m^2s^{-1}\times10^{-10}$.

Table S7. Influence of the sample stability on diffusion coefficient and resolution.

Time(h)	reserpine	yohimbine	catharanthine	β-carboline
**1h**	5.22	2.93	2.70	2.21
**2h**	5.21	2.94	2.58	2.21
**4h**	5.21	2.93	2.60	2.19
**8h**	5.22	2.99	2.63	2.21

$\Delta D_1 = D_{yohimbine} - D_{reserpine}$, $\Delta D_2 = D_{catharanthine} - D_{yohimbine}$, $\Delta D_3 = D_{\beta\text{-carboline}} - D_{catharanthine}$

Diffusion coefficient (D) was reported in $m^2s^{-1}\times10^{-10}$.

The literature indicates that several types of databases can be used. In the previous work of this research group, the ^{1}H chemical shift data of natural products was collected from the literature and has been used to establish a large "experimental database". More than 200 indole alkaloids representing *Apocynaceae* were included in this experimental database. These alkaloids were selected because of the diversity of their structures. For some compounds whose ^{1}H spectra were not recorded in the literature, we used the NMRP module in the MestReNova software to make predictions in order to enrich the database.

As a routine verification test, the MestReNova software was used to search ^{1}H spectrum extracted from the DOSY spectrum of the *Alstonia mairei* mixtures and ranked by accuracy. In order to make the results more reliable, we used the search result of the proton spectrum extracted from the model mixture with SDS as a control. The option with the highest score was considered to be the compound we separated. Compared with the score of the experiment without SDS, it is obvious that the addition of a matrice can improve the resolution of DOSY spectra. The analyte signals were mixed together in the DOSY spectrum due to the similar structures of analytes. In practical sample, it can be seen that, the signal overlap was serious without SDS, and we can only roughly extract four ^{1}H spectra from the spectra. After adding a matrix, the strong overlapping areas can be well separated, we got nine ^{1}H spectra. Therefore, the matrice can be added to make the search results more accurate. And the results are as follows:

Figure S3. (a) DOSY spectrum of mixtures (each component was 2 mg) dissolved in 600 μL of DMSO-d_6. (b) DOSY spectrum of the same mixtures as in (a), but in the presence of 2 mg SDS.

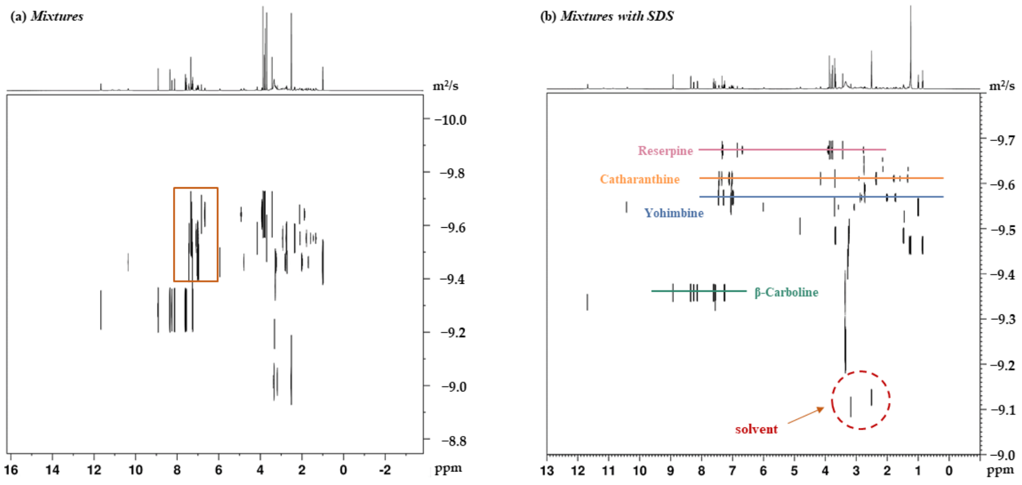

Table S8. Searching ^{1}H spectrum extracted from the DOSY spectrum of the model mixture in the database, and the following are the options with the highest scores and their corresponding compounds.

(a) Scores obtained from model mixtures without SDS and the corresponding compounds.

**Spectra**	**Compound**	**Score (‰)**
**C1**	Reserpine	858
**C2**	Yohimbine	756
**C3**	Catharanthine	616
**C4**	β-Carboline	787

(b) Scores obtained from model mixtures with the addition of SDS and the corresponding compounds.

**Spectra**	**Compound**	**Score (‰)**
**C1**	Reserpine	923
**C2**	Yohimbine	953
**C3**	Catharanthine	989
**C4**	β-Carboline	998

Figure S4. DOSY spectra of a mixture of the real total alkaloid extract (20 mg) before (a, upper) and after (b, lower) the addition of SDS (10 mg) in DMSO-d6 (600 μL). Experimental temperature was 298 K.

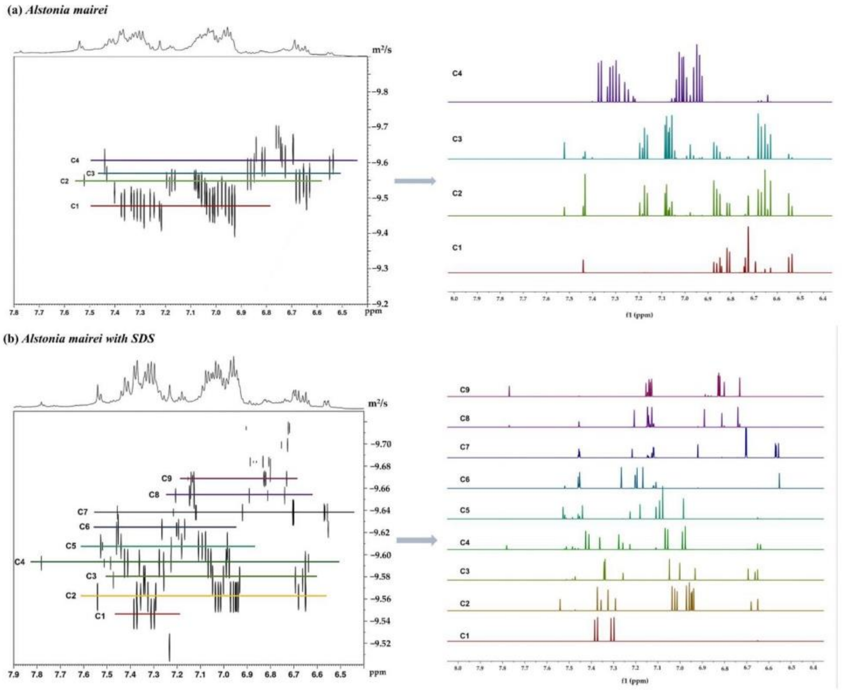

Table S9. Searching ^{1}H spectrum extracted from the DOSY spectrum of the actual sample mixture in the database, and the following are the options with the highest scores and their corresponding compounds.

(a) Search scores of four ^{1}H spectra extracted from total alkaloid extract of *Alstonia Mairei* without SDS in the database and the corresponding compounds.

Spectra	Compound	Score (‰)
**C1**	Syringaresino	724
**C2**	Echitoveniline	538
**C3**	10-Hydroxy-dihydroperaksine	673
**C4**	N1-(hydroxymethyl)isositsirikine	848

(b) Search scores of nine ^{1}H spectra extracted from total alkaloid extract of *Alstonia Mairei* with SDS in the database and the corresponding compounds.

Spectra	Compound	Score (‰)
**C1**	Tueiaoine	833
**C2**	N1-(hydroxymethyl)isositsirikine	898
**C3**	Rauvotetraphylline B	894
**C4**	10-Hydroxy-dihydroperaksine	929
**C5**	10-methoxypanarine	901
**C6**	Echitoveniline	868
**C7**	Syringaresino	870
**C8**	17-acetylsandwicine	848
**C9**	Cyclodicaprylactam	808
